# Candesartan Normalizes Changes in Retinal Blood Flow and p22phox in the Diabetic Rat Retina

**DOI:** 10.3390/pathophysiology28010008

**Published:** 2021-03-02

**Authors:** Randa S. Eshaq, Megan N. Watts, Patsy R. Carter, Wendy Leskova, Tak Yee Aw, Jonathan Steven Alexander, Norman R. Harris

**Affiliations:** Department of Molecular & Cellular Physiology, Louisiana State University Health Sciences Center in Shreveport, Shreveport, LA 71130, USA; reshaq@lsuhsc.edu (R.S.E.); mwatt1@lsuhsc.edu (M.N.W.); deaccarter51@yahoo.com (P.R.C.); wzhang@lsuhsc.edu (W.L.); taw@lsuhsc.edu (T.Y.A.); jalexa@lsuhsc.edu (J.S.A.)

**Keywords:** diabetes, retina, angiotensin II, candesartan, perfusion, superoxide

## Abstract

Angiotensin II has been implicated in the progression of diabetic retinopathy, which is characterized by altered microvasculature, oxidative stress, and neuronal dysfunction. The signaling induced by angiotensin II can occur not only via receptor-mediated calcium release that causes vascular constriction, but also through a pathway whereby angiotensin II activates NADPH oxidase to elicit the formation of reactive oxygen species (ROS). In the current study, we administered the angiotensin II receptor antagonist candesartan (or vehicle, in untreated animals) in a rat model of type 1 diabetes in which hyperglycemia was induced by injection of streptozotocin (STZ). Eight weeks after the STZ injection, untreated diabetic rats were found to have a significant increase in tissue levels of angiotensin converting enzyme (ACE; *p* < 0.05) compared to non-diabetic controls, a 33% decrease in retinal blood flow rate (*p* < 0.001), and a dramatic increase in p22phox (a subunit of the NADPH oxidase). The decrease in retinal blood flow, and the increases in retinal ACE and p22phox in the diabetic rats, were all significantly attenuated (*p* < 0.05) by the administration of candesartan in drinking water within one week. Neither STZ nor candesartan induced any changes in tissue levels of superoxide dismutase (SOD-1), 4-hydroxynonenal (4-HNE), or nitrotyrosine. We conclude that one additional benefit of candesartan (and other angiotensin II antagonists) may be to normalize retinal blood flow, which may have clinical benefits in diabetic retinopathy.

## 1. Introduction

Diabetic retinopathy is characterized by microvascular pathology, including an early decrease in retinal blood flow rate, the development of capillary occlusions, and localized regions of ischemia and hypoxia. Perhaps in response to these vascular changes, a later phase of neovascularization leading to poorly controlled microvascular function occurs, with the new and leaky blood vessels interfering with light transmission to the photoreceptors.

The production of reactive oxygen species (ROS) also occurs early in the progression of experimental diabetic retinopathy, with associated pathological consequences on the retinal neurons. However, the time course of ROS production may not coincide precisely with the changes in retinal perfusion, indicating that the pathways may be either independent or sequential. We have found that retinal blood flow decreases significantly in rat and mouse models of type 1 diabetes as early as 3–4 weeks [1,2,3], and at least in diabetic mice, appears to remain decreased for another 5 months [4]. Many studies have shown that substantial oxidative and nitrosative stress also develops within the first 6 months in diabetic rats and mice; however, the extent to which the oxidative effects occur in the first two months (at which time retinal blood flow is initially reduced) is debatable given relatively unchanged levels of glutathione, superoxide dismutase (SOD), catalase, NADPH oxidase, and malondialdehyde (a marker of lipid peroxidation) [5,6,7,8,9,10,11,12].

Angiotensin II is a vasoconstrictor that not only has been implicated in the reduction of retinal blood flow in models of diabetes, but also has been found to increase oxidative stress and expression levels of the superoxide-generating enzyme NADPH oxidase. Therefore, inhibitors of angiotensin II could have a two-fold benefit of not only improving blood flow in the early phase of diabetes, but also limiting oxidative stress (if present). Accordingly, in the current study, we evaluated both potential benefits by administering the angiotensin II receptor antagonist candesartan to rats during the final week of an 8-week diabetes protocol, with measurements of retinal blood flow and parameters related to the potential development of oxidative and/or nitrosative stress.

## 2. Materials and Methods

### 2.1. Animal Groups

Male Wistar rats (Harlan Laboratories, Houston, TX, USA) at an age of ~2 months were injected on 3 consecutive days with 30 mg/kg i.p. streptozotocin (STZ; Sigma-Aldrich, St. Louis, MO, USA) within 15 min of being dissolved in pH 4.5 sodium citrate buffer. Age-matched non-diabetic controls were injected with sodium citrate buffer alone. Body weights and nonfasting plasma glucose values (AlphaTRAK glucometer; Abbott Laboratories, Abbott Park, IL, USA) were obtained on the day of injection and again eight weeks later, on the day of the remaining experimental measures. STZ-injected rats with glucose values < 300 mg/dL were considered nondiabetic and were excluded from the study. No insulin was given during the protocol. Approximately one-half of the rats (20/42 nondiabetic; 19/37 diabetic) were administered the angiotensin II receptor antagonist candesartan in their drinking water during the final week of the 8-week protocol. The experimental protocols were approved by the Institutional Animal Care and Use Committee of LSUHSC-S and performed according to the criteria outlined by the National Institutes of Health.

### 2.2. Microsphere Measurement of Retinal Blood Flow

A subset of the rats (N = 9–11 per group) was used for measurements of retinal blood flow using microspheres, as we have described previously [13], and in accordance with the optimized procedure developed by Wang et al. [14,15]. The animals were anesthetized with 100 mg/kg ketamine and 40 mg/kg pentobarbital, prior to the cannulation of the right femoral artery. An incision was made into the upper abdomen, which allowed the insertion of a 27-gauge needle through the diaphragm into the left ventricle. Fluorescent microspheres (2.5 × 10^6^, 8 μm diameter; Bangs Laboratories, Fisher, IN, USA) were injected into the left ventricle while a reference blood sample was collected through the cannulated femoral artery. The rate of arterial withdrawal began 10 s before the injection and continued for a period of 30 s following the injection. The eyes were removed (and the rat euthanized with an overdose of pentobarbital), and the retinas of both eyes were prepared as flat mounts for counting the number of microspheres lodged in the retinal microvessels. The number of microspheres in the reference blood sample was counted with the use of a hemacytometer, and retinal blood flow rate was calculated as the femoral blood withdrawal rate multiplied by the ratio of microspheres found in the retinal tissue and divided by the number counted in the blood sample.

### 2.3. Retinal Tissue Western Blots

In a subset of rats (N = 9–15 per group), the eyes were removed following anesthesia with 100 mg/kg ketamine and 40 mg/kg pentobarbital, just prior to euthanasia with an overdose of pentobarbital. The retinas were collected into 250 μL ice-cold radio-immuno-precipitation-assay (RIPA) buffer with protease inhibitors and immediately put on ice. The tissue was homogenized for 10 s on ice, then centrifuged for 10 min at 10,000× *g* and 4 °C. The supernatant was collected for measurement of total protein concentration (using a bicinchoninic acid kit), and 25 μg of protein were loaded into a 4–20% gradient gel, run for 1 h and the protein then transferred to a nitrocellulose membrane for 1 h, at 100 V and room temperature. Membranes were then blocked using a protein-free block (Pierce, Rockford, IL, USA) at room temperature for 1 h. Primary antibodies diluted in the protein-free block were incubated overnight, with secondary antibodies incubated afterward for 1 h. The blots were developed using a Pierce enhanced chemiluminescence kit (Pierce, Rockford, IL, USA), with protein intensities normalized to staining for β-actin. Table 1 provides additional details for the individual Western blot assays. Antibodies were obtained from EMD Millipore (Billerica, MA, USA), Cell Signaling Technology (Danvers, MA, USA), Abcam Antibodies (Cambridge, MA, USA), Origene (Rockville, MD, USA), and Santa Cruz Biotechnology (Santa Cruz, CA, USA). Specificity was confirmed with the use of non-immune isotype sera and by omitting the primary antibody.

### 2.4. Statistics

Newman-Keuls post-hoc multiple comparison tests following ANOVA (GraphPad Prism, La Jolla, CA, USA) were used to identify statistical differences between groups, using the criterion of *p* < 0.05. Data are presented as means ± standard error.

## 3. Results

### 3.1. Glucose Levels and Body Weights of Experimental Animals

Table 2 provides the body weights and plasma glucose values for the four groups of rats, with hyperglycemia accompanied by a slower rate of weight gain in the diabetic rats, as expected. A subset of the body weight and plasma glucose data from the untreated rats in Table 2 has been published previously in a separate investigation [13].

### 3.2. Retinal Blood Flow Rate

**Figure 1:**
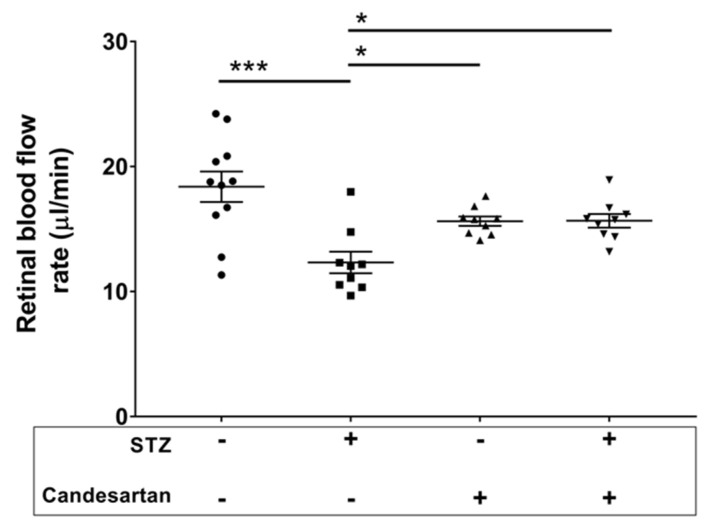
Retinal blood flow rates in the four groups of rats: untreated non-diabetic controls, untreated STZ, candesartan-treated non-diabetic controls, and candesartan-treated STZ. Candesartan treatment in the STZ rats was able to attenuate the STZ-induced decrease in retinal blood flow rate. N = 9–11 per group, * *p* < 0.05 and *** *p* < 0.001 between indicated groups.

Eight weeks of diabetes resulted in a 33% drop in retinal blood flow rate (*p* < 0.001), as measured by the microsphere infusion technique, with this decrease attenuated significantly (*p* < 0.05) by the administration of candesartan (Figure 1).

### 3.3. Angiotensin Pathway

**Figure 2:**
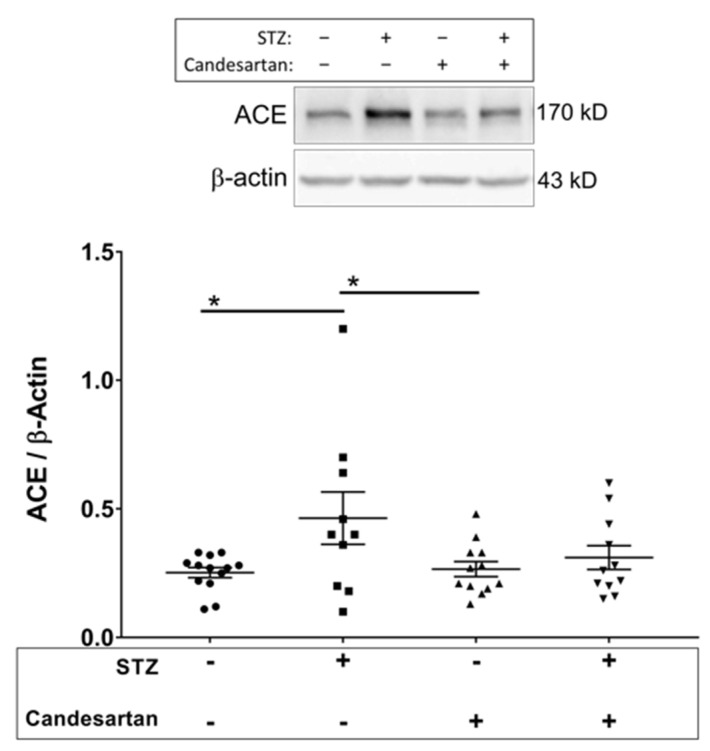
Retinal tissue protein levels (Western blot data; normalized to β-actin) of angiotensin converting enzyme (ACE) in the four groups of rats: untreated non-diabetic controls, untreated STZ, candesartan-treated non-diabetic controls, and candesartan-treated STZ. ACE levels were increased in the diabetic rats, but only in the absence of candesartan treatment. N = 10–13 per group, * *p* < 0.05 between indicated groups.

Angiotensin II is produced from angiotensin I by angiotensin converting enzyme (ACE). The retinal levels of ACE were significantly higher (*p* < 0.05) in diabetic rats compared to non-diabetic controls (Figure 2); however, no increase in ACE was observed in diabetic rats treated with candesartan.

**Figure 3:**
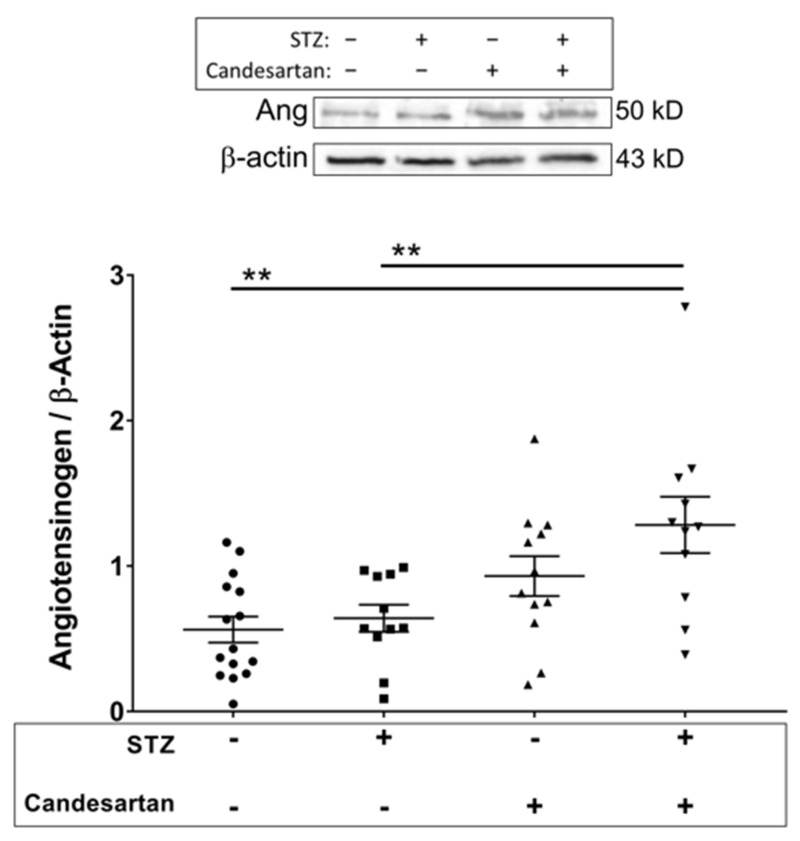
Retinal tissue protein levels (Western blot data; normalized to β-actin) of angiotensinogen in the four groups of rats: untreated non-diabetic controls, untreated STZ, candesartan-treated non-diabetic controls, and candesartan-treated STZ. Angiotensinogen levels were increased in the diabetic rats only with the administration of candesartan. N = 11–15 per group, ** *p* < 0.01 between indicated groups.

Angiotensin I is produced via the renin-mediated conversion of angiotensinogen, the levels of which are shown in Figure 3 for the four groups.

**Figure 4:**
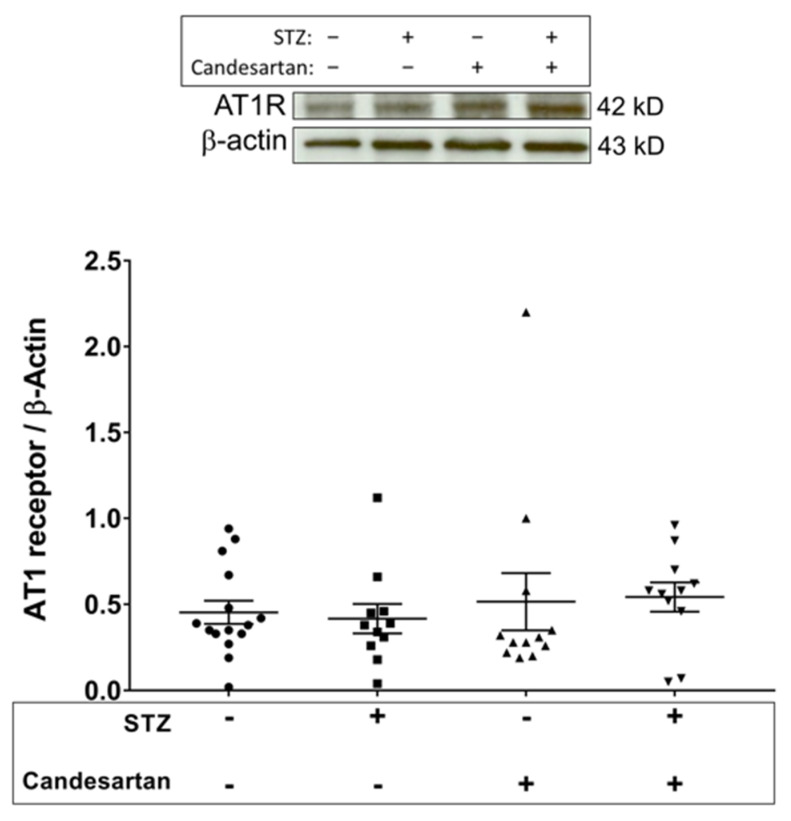
Retinal tissue protein levels (Western blot data; normalized to β-actin) of the angiotensin II type 1 receptor (AT1) in the four groups of rats: untreated non-diabetic controls, untreated STZ, candesartan-treated non-diabetic controls, and candesartan-treated STZ. No statistical differences were found between groups. N = 11–15 per group.

No change in angiotensinogen was induced by diabetes alone; however, administration of candesartan to diabetic rats resulted in a significant increase of angiotensinogen. No differences between groups were found for retinal levels of the angiotensin II type 1 receptor (Figure 4).

### 3.4. Oxidative and Nitrosative Stress Markers

**Figure 5:**
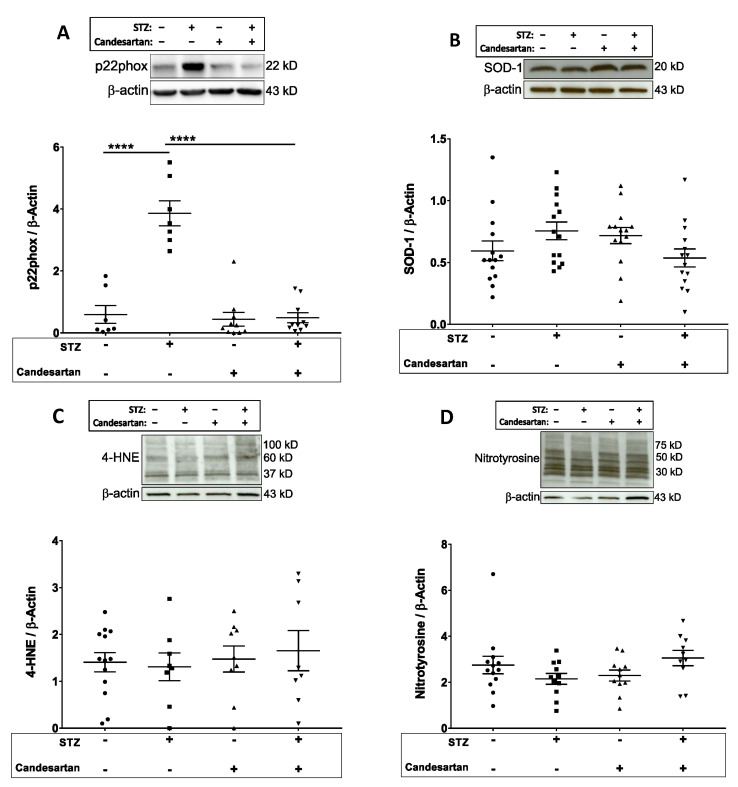
Retinal tissue protein levels (Western blot data; normalized to β-actin) of (**A**) the p22phox subunit of NADPH oxidase, (**B**) SOD-1, (**C**) 4-HNE, and (**D**) nitrotyrosine in the four groups of rats: untreated non-diabetic controls, untreated STZ, candesartan-treated non-diabetic controls, and candesartan-treated STZ. A significant increase (*p* < 0.05) in p22phox was observed in the diabetic retina, which was attenuated with candesartan treatment; however, no statistical differences in SOD-1, 4-HNE, or nitrotyrosine were found between groups. N = 9–14 per group, **** *p* < 0.0001.

Expression of NADPH oxidase was evaluated by the levels of the p22phox subunit of the enzyme. The retinal Western blot intensities of p22phox were increased 5-fold (*p* < 0.05) in the diabetic rats when compared to controls (Figure 5A), with this increase attenuated by the administration of candesartan. However, neither STZ nor candesartan significantly affected retinal levels of the antioxidant superoxide dismutase (SOD-1; Figure 5B), or the 4-HNE or nitrotyrosine measures of oxidative/nitrosative stress (Figure 5C,D).

## 4. Discussion and Conclusions

The results of this study demonstrate that the diabetes-induced decrease in retinal blood flow can be alleviated by the administration of the angiotensin II receptor antagonist candesartan. The influence of angiotensin II could occur not only via receptor-mediated calcium release that causes vascular constriction, but also through activation of NADPH oxidase to elicit the formation of reactive oxygen species (ROS). However, even though the levels of p22phox, an essential subunit of the NADH/NADPH superoxide-generating enzyme, were significantly increased in the diabetic retina, no significant alterations in measures of oxidative stress were observed with either short-term diabetes or angiotensin II receptor blockade.

The 33% decrease in retinal blood flow that we measured in STZ rats is similar to the 30–35% decrease found in the early stages of human diabetic retinopathy [16,17,18]. The instigation and mechanisms of the early diabetes-induced decrease in retinal blood flow have yet to be fully determined, although a previous study in rats has shown that ACE inhibition and angiotensin II receptor blockade is effective in restoring retinal blood flow at the 2-week time point following STZ injection in rats [19]. The experiments of the current study extend this time course and indicate that angiotensin II blockade attenuates decreases in retinal blood flow through 8 weeks of STZ-induced diabetes.

It has been speculated that the early decrease in retinal blood flow could be a response to a decreased rate of metabolism in the retina [20,21], in which a decrease in oxygen utilization may be matched by a decrease in oxygen delivery. Reports indicate that a portion of retinal neurons (especially oxygen-consuming photoreceptors) become apoptotic and die early in diabetes [22,23,24,25]; moreover, other diabetes-related changes in the retina may lead to a decrease in energy demand, [26,27], with a resulting decrease in oxygen consumption [28,29,30].

With a decrease in oxygen consumption, retinal oxygen levels could rise significantly higher than normal, with the excess oxygen potentially converted to reactive oxygen species by neurons [31,32]. Therefore, vasoconstriction of the retinal vessels could be viewed as a protective mechanism against oxidative stress, although this protection could come at the increased risk of capillary occlusions occurring as a result of decreased perfusion pressure downstream of constricting arterioles. Therefore, any attempts to override the vasoconstriction that occurs early in the diabetic retina could result in hyperoxygenation and enhanced production of oxygen radicals. Moreover, the minimal changes in several measures of oxidative stress in this study, and in other studies of the first 4–8 weeks of the diabetic retina in rats [5,6,7,8,9,10,11,12], could be influenced, at least partially, by the reduced supply of oxygen being delivered to the retina, although this speculation needs further investigation. Given this hypothesized scenario, the administration of candesartan, which significantly improves retinal perfusion, could potentially contribute to the development of oxidative stress via excess oxygen delivery. However, inasmuch as angiotensin II has been found to increase the expression of NADPH oxidase and contribute to the formation of superoxide, any such contribution to oxidative stress by improved oxygen delivery with candesartan would be potentially neutralized.

A second pathway of the renin-angiotensin system is the ACE2-angiotensin(1–7)-Mas receptor (MasR) axis. The cleavage of one amino acid from angiotensin II by ACE2 [33] leads to the formation of angiotensin(1–7), which in turn exerts its effects via its interaction with MasR [34]. Angiotensin(1–7) has been shown to counteract the effects of angiotensin II, and promote its antihypertensive actions via its vasodilatory, antiproliferative, antithrombotic, and antiarrythmogenic effects [35]. The observed candesartan-induced restoration of blood flow in the diabetic retina, which was accompanied by an increase in angiotensinogen levels, might be explained by an increase in angiotensin(1–7). The blockade of angiotensinogen processing via ACE may elevate levels of angiotensinogen which facilitates the production of angiotensin(1–7) and angiotensin(1–9) via ACE2. In a previous study, the blockade of ACE resulted in a decrease in retinal angiotensin II levels, and an increase in angiotensin(1–7) levels in diabetic rats [36], which could be explained by the increased conversion of angiotensin II to angiotensin(1–7) by ACE2. Future validations could be accomplished with ACE activity measurements as well as ELISA evaluation of circulating angiotensin II. The angiotensin II receptor antagonist candesartan decreased levels of ACE in STZ induced diabetic rats, which was unexpected. This could reflect a diversion of the processing pathway to increase angiotensin I which yields angiotensin(1–9) and angiotensin(1–7), an effect that reflects elevated ACE2 levels [37].

In our study, we observed a substantial diabetes-induced increase in the expression of p22phox (a subunit of NADPH oxidase). Due to the nature of variable susceptibilities to degradation of ROS in different compartments, p22phox was used as an indirect method of assessing the potential for ROS generation in the retina. Even though not a direct measure of ROS, this measurement has been utilized in other studies where p22phox levels have been used to take advantage of the stable nature of this ROS-generating component as indirect evidence of the potential for ROS development in the eye [38]. Additionally, other investigators have presented a case for a significant role for NADPH in the oxidative stress associated with diabetic retinopathy [39,40,41]. Our data partially support this possibility, in that an increase in the levels of p22phox may lead to an increased production of superoxide. However, it should be noted that p22phox levels are not a direct measure of NADPH oxidase activity nor ROS levels, and therefore, the limitations of this measurement are acknowledged.

Lee et al. [8,9], using the same time point (8 weeks) of STZ-induced diabetes, found a decrease in immunostaining of the retina for NADPH oxidase, but despite this decrease, they found that frozen cross-sections of the retina demonstrated increased superoxide-mediated conversion of dihydroethidium. Their interpretation of these results was that the excess superoxide in the cross-sections was being generated by a different pathway than via NADPH oxidase. Other sources of superoxide include the mitochondrial production of superoxide, which is thought to induce a subsequent increase in other superoxide-generating pathways such as uncoupled nitric oxide synthase, xanthine oxidase, and redox changes [42]. An increase in superoxide also could result from a decrease in superoxide dismutase (SOD), although our study and others [7,10] have not found a decrease in either copper-zinc SOD (SOD-1) or manganese SOD (SOD-2) in the first 2 months of STZ-induced diabetes. However, it should be mentioned that contrasting results are provided by Obrosova et al. [5,6] with statistically significant decreases in SOD found in the STZ rat retina at a very similar time point of 6 weeks.

Another scavenger of superoxide is glutathione (GSH), with a decrease in GSH potentially leading to an excess of superoxide and oxidative stress. However, one of our studies [12], and reports by others [5,6,11], find no decrease in retinal GSH at the 6–8 week time point of STZ-induced diabetes (in contrast to the decrease reported by Ola et al. [43]). Our observed lack of changes in retinal 4-HNE and nitrotyrosine at 8-weeks STZ, and the lack of increase in retinal malondialdehyde at a similar time point of 6-weeks STZ [6], are consistent with minimal early increases in oxidative and/or nitrosative stress in this rat model of diabetic retinopathy.

At later time points in the rat STZ model, however, retinal oxidative stress becomes much more substantial and consistent among various investigators. GSH is reported to decrease by ~50% by 3 months [44], and then by ~75–80% by 6 months [45]. Malondialdehyde increases two-fold by 3–4 months [44,46], with Si et al. reporting a doubling of retinal NADPH oxidase, a 4-fold increase in superoxide production, a three-fold increase in peroxynitrite formation, and a 50% reduction in SOD at 4 months in the rat STZ model [46]. A final consideration is that at least two rat STZ studies have found a role for angiotensin II in the development of retinal oxidative stress [43,47]. This role was not apparent in our investigation; however, we cannot exclude the possibility of local (vascular) ROS generation that could go undetected by our measurements.

In summary, at an early time point of 8-weeks diabetes in rats, candesartan can successfully attenuate the early angiotensin-mediated decrease in retinal blood flow and the diabetes-induced increase in the levels of retinal p22phox, despite having a minimal effect on oxidative stress, which in itself was not substantially enhanced in this model. The literature suggests that models using longer durations of diabetes may produce a greater extent of retinal oxidative stress, with the role of angiotensin II in that pathway yet to be determined.

## Figures and Tables

**Table 1:** Western blot antibodies and dilutions.

	Primary Antibody			Secondary Antibody		
	Host	Dilution	Product		Dilution	Product
Angiotensin converting enzyme	mouse	1:5000	Millipore MAB 4051	Goat anti-mouse	1:10,000	Invitrogen G21040
Angiotensinogen	goat	1:2000	Santa Cruz SC-7419	Donkey anti-goat	1:10,000	Santa Cruz SC-2020
Angiotensin II type 1 receptor	mouse	1:1000	Abcam ab9391	Goat anti-mouse	1:10,000	Invitrogen G21040
Nitrotyrosine	rabbit	1:15,000	Millipore 06-284	Goat anti-rabbit	1:10,000	Invitrogen G21234
Superoxide dismutase-1	rabbit	1:15,000	Origene TA300916	Goat anti-rabbit	1:10,000	Invitrogen G21234
4-hydroxynonenal	rabbit	1:1000	Abcam ab45545	Goat anti-rabbit	1:10,000	Invitrogen G21234
p22phox	rabbit	1:500	Santa Cruz SC-20781	Goat anti-rabbit	1:10,000	Invitrogen G21234

**Table 2:** Body weight and plasma glucose values.

		Initial Body Weight (g)	Final Body Weight (g)	Initial Plasma Glucose (mg/dL)	Final Plasma Glucose (mg/dL)
Untreated Controls	N = 22	147 ± 4	461 ± 9	151 ± 6	160 ± 4
Untreated STZ	N = 18	139 ± 4	276 ± 10 ***	146 ± 5	529 ± 23 ***
Candesartan Controls	N = 20	136 ± 4	445 ± 6	157 ± 6	155 ± 7
Candesartan STZ	N = 19	138 ± 5	287 ± 13 ***	155 ± 6	574 ± 27 ***

*** *p* < 0.001 vs. untreated controls and candesartan controls.

## Data Availability

Data sharing not applicable.
